# Innovative Curved-Tip Reactor for Non-Thermal Plasma and Plasma-Treated Water Generation: Synergistic Impact Comparison with Sodium Hypochlorite in Dental Root Canal Disinfection

**DOI:** 10.3390/ma16227204

**Published:** 2023-11-17

**Authors:** Raúl Arguello-Sánchez, Régulo López-Callejas, Benjamín Gonzalo Rodríguez-Méndez, Rogelio Scougall-Vilchis, Ulises Velázquez-Enríquez, Antonio Mercado-Cabrera, Rosendo Peña-Eguiluz, Raúl Valencia-Alvarado, Carlo Eduardo Medina-Solís

**Affiliations:** 1 Dental Reseach Center and Advanced Studies “Dr. Keisaburo Miyata”, School of Dentistry, Autonomous University of Mexico State, Av. Paseo Tollocan, 13 Universidad, Toluca de Lerdo 50130, Mexico; rarguellos@uaemex.mx (R.A.-S.); rscougallv@uaemex.mx (R.S.-V.); uvelazqueze@uaemex.mx (U.V.-E.); cemedinas@uaeh.edu.mx (C.E.M.-S.); 2 Department of Physics, National Institute for Nuclear Research, Carretera Mexico-Toluca S/N, Ocoyoacac 52750, Mexico; regulo.lopez@inin.gob.mx (R.L.-C.); antonio.mercado@inin.gob.mx (A.M.-C.); rosendo.eguiluz@inin.gob.mx (R.P.-E.); raul.valencia@inin.gob.mx (R.V.-A.); 3 Dentistry Academic Area of the Health Sciences Institute, Autonomous University of Hidalgo State, Exhacienda de la Concepción S/N Carretera Actopan-Tilcuautla, San Agustin Tlaxiaca 42160, Mexico

**Keywords:** non-thermal plasma, plasma-treated water, veneering, ceramic, bacteria, root canals

## Abstract

Non-thermal plasmas (NTPs), known as cold atmospheric plasmas (CAPs), hold great potential for diverse medical applications, including dentistry. However, traditional linear and rigid dielectric barrier discharge reactors used for NTP generation encounter limitations in accessing oral cavities and root canals. To address this issue, we have developed an innovative NTP reactor featuring an angled end for improved accessibility. The central copper electrode, with a 0.59 mm diameter and adjustable length for desired angulation, is coated with zircon powder (ZrSiO_4_) to ensure stable NTP generation. This central electrode is housed within a stainless steel tube (3 mm internal diameter, 8 mm external diameter, and 100 mm length) with a 27° angle at one end, making it ergonomically suitable for oral applications. NTP generation involves polarizing the reactor electrodes with 13.56 MHz radio frequency signals, using helium gas as a working medium. We introduce plasma-treated water (PTW) as an adjunctive therapy to enhance biofilm eradication within root canals. A synergistic approach combining NTP and PTW is employed and compared to the gold standard (sodium hypochlorite, NaOCl), effectively neutralizing *Enterococcus faecalis* bacteria, even in scenarios involving biofilms. Moreover, applying NTP in both gaseous and liquid environments successfully achieves bacterial inactivation at varying treatment durations, demonstrating the device’s suitability for medical use in treating root canal biofilms. The proposed NTP reactor, characterized by its innovative design, offers a practical and specific approach to plasma treatment in dental applications. It holds promise in combatting bacterial infections in root canals and oral cavities.

## 1. Introduction

Bacteria lodged in the root canal cause pulpitis and apical periodontitis. Once these bacteria organize themselves into a biofilm, dental debridement becomes difficult due to their ability to adhere to and colonize the root canal, where they develop and secrete an exopolysaccharide that acts as an adhesion and protection matrix for the bacteria, making them more resistant to antimicrobial agents than in planktonic bacteria [1,2,3]. The purpose of endodontics is to eliminate the presence of intraradicular biofilm since it is considered an important factor in the pathogenesis of endodontic infections, and its total elimination is essential for the success of endodontic treatment; this can be achieved through mechanical instrumentation of the root canal and irrigation with antiseptic solutions [4,5].

The gold standard for dental disinfection is chemomechanical treatment, which is carried out with manual or assisted rotary instruments and using chemical solutions such as sodium hypochlorite (NaOCl) [6,7,8]. The leading causes of endodontic failure are the infection of the dentinal tubules and/or lateral canals, anatomical complexity, fractures or fissures, and extraradicular biofilm [9]. This has led to the search for alternative disinfection methods to avoid endodontic failure. One of these alternatives could be the application of non-thermal plasma (NTP) or cold atmospheric plasma (CAP).

NTP is excellent for wound disinfection, sterilization, and healing in biomedical applications. This is possible because when generating the NTP and depending on the gas used, reactive species, charged particles, and UV photons can be produced, which have a high oxidative potential and, therefore, can attack and disrupt the molecular structures of microorganisms [10]. Likewise, the surfaces of medical devices can be optimized to improve their biocompatibility and biofunctionality [11,12].

The NTP is generated by providing energy to a gas using an electric field, which achieves its ionization. An NTP is composed of ions, free electrons, and atoms in their elementary or excited state. The electric field causes the dissociation of the gas atoms or molecules into ions and electrons. Subsequently, the atoms or molecules in the excited state lose their energy by colliding with other particles or by the emission of photons [13]. The NTP is formed at atmospheric pressure in frequency ranges of the order of units from kHz to MHz or in microwaves (GHz), with voltages up to 100 kV [14].

Another way to achieve bacterial inactivation is by utilizing water interacting with NTP, a process known as Plasma-Treated Water (PTW). The application of PTW has garnered significant interest due to its potential environmental, medical, and even food technology applications, among others. The primary technique for producing PTW involves generating plasma either outside or within the water to produce ions that result in various highly reactive oxygen species (ROS) and nitrogen species (RNS), which are essential for bacterial inactivation. One advantage of PTW is its ability to be applied rapidly and easily, regardless of the surface’s form. Furthermore, this innovative approach opens new avenues for enhancing disinfection and treatment strategies, contributing to various fields, including public health, agriculture, and industrial processes [15].

Plasma can be generated through various methods, with some requiring high vacuum systems. Examples include glow discharge, microwave discharge, inductively coupled discharge, and capacitively coupled discharge. However, when operating at atmospheric pressure, NTP can be generated using corona discharge and dielectric barrier discharge (DBD). Notably, these methods eliminate the need for high-vacuum systems [16,17].

In the setup of a DBD-type plasma between two electrodes where the discharge occurs, one or both can be coated with a dielectric material [18,19]. The DBD forms when an alternating voltage or pulsed voltage is applied across these electrically insulated electrodes, leading to an accumulation of charge on the surface of the dielectric. This sequence of events results in multiple discharges in intervals of microseconds, which supplies the necessary ionization level to sustain a plasma discharge. The plasma thus created can be maintained as long as the energy supplied is adequate to counteract the recombination process; otherwise, the plasma discharge will cease to exist [20,21,22].

An electric field is also established between electrically insulated electrodes, and if the field strength is sufficient, an electric discharge occurs. However, the insulation prevents the generation of significant current flow, preventing the gas from heating. Instead, the electrons migrate toward the positive electrode but cannot escape due to insulation. In a minimal time, the resulting charge compensates for the high-voltage field created, extinguishing the discharge [19,20].

Various DBD reactor designs have been described [19]. The characteristics of DBD plasma reactors mainly depend on the electrical parameters of the power supply, the chosen geometry, and the properties of the dielectric barrier material. Studies have concluded that three dielectric characteristics are of great importance, the type of dielectric material, the thickness of the dielectric barrier, and the surface roughness of the dielectric barrier, as they impact the discharge mode and its emission spectrum [23,24].

Indeed, some reviews focus on dielectric materials and their influence on the performance of the DBD plasma reactor, considering factors like thickness [18,24,25]. Researchers have also investigated the relationship between the concentration of discharge filaments and the subsequent dielectric breakdown. Additionally, studies have explored the surface temperature of the dielectric and its effect on the transition from glow discharge to filamentary discharge. Notably, it has been demonstrated that certain ceramics, known for their corrosion resistance in both high- and low-temperature conditions, exhibit excellent dielectric properties and heat conduction [24].

Ceramic materials serve specific purposes based on their application. Multifunctional ceramics consist of different materials, each contributing a distinct function. Some of the most studied ceramics include aluminium oxide doped with MgO [26], calcium zirconate oxide doped with MgO [24], and yttrium-stabilized zirconia [27]. These compositions have been extensively investigated for their unique properties and potential applications.

The reactors that generate NTP for biomedical applications need specific characteristics, including being manageable and ergonomic. However, conventional NTP reactors are often rigid and straight, making them unsuitable for direct use in oral cavity root canals. A jet-type reactor with a curved terminal has been developed to overcome this limitation [28,29]. These reactors can operate with peak voltages of up to 18 kV and working frequencies between 20 and 50 kHz. The curved design allows for easier insertion and application of NTP in root canals, making it more practical for dental treatments.

Another type of reactor used for NTP generation is the Thorns plasma reactor [30], which creates a plasma in pre-established radial directions. It operates with voltages around 16 kV and frequencies of 10 kHz, and the diameter of the tip is 6 mm. Although it can be inserted into the root canal for treatment, the tip size may make it impractical in some instances where the canal’s dimensions are too narrow. This limitation could restrict its applicability in specific dental procedures.

This study introduces an innovative approach for addressing biofilms within root canals. The primary objective is the efficient elimination of these biofilms by utilizing a novel curved-tip reactor designed for NTP generation. This unique reactor was also employed for PTW production. Both processes were compared to the conventional gold standard in root canal treatments involving sodium hypochlorite. In vitro assessments demonstrated the effectiveness of bacterial and biofilm removal from dentin samples. The curved reactor design enhances manoeuvrability and facilitates application in challenging-to-access regions, potentially offering an improved treatment modality for endodontic infections.

## 2. Experimental Setup

### 2.1. Non-Thermal Plasma Reactor Design with a Curved Terminal

**Figure 1:**
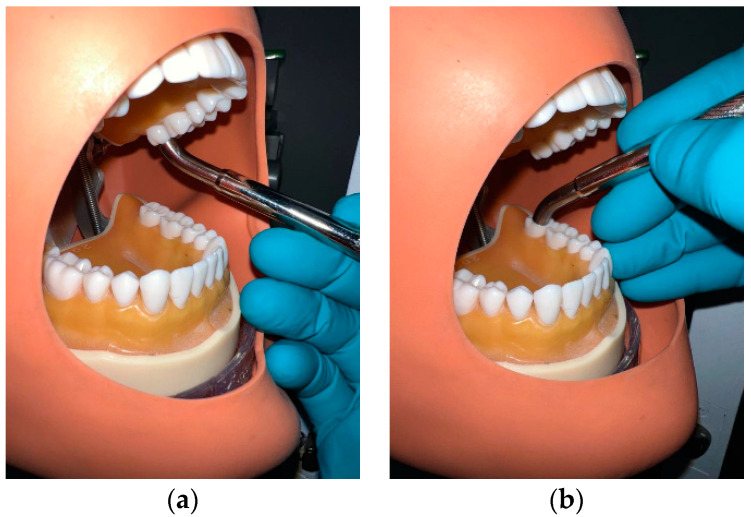
Placement of the NTP-generating reactor inside the mouth, illustrating its application in the most profound areas: (**a**) upper molar and (**b**) lower molar.

The NTP-generating reactor proposed in this study features a coaxial design with a 27° angle at one end. This configuration allows for convenient insertion into the oral cavity and precise positioning to apply NTP from the incisors to the lower and upper molars (refer to Figure 1). Consequently, the plasma beam is directed perpendicular to the target area, ensuring accurate and targeted treatment for optimal therapeutic outcomes.

The reactor has specific characteristics, including an internal electrode length of 99 mm and an external electrode length of 100 mm. Both electrodes have an angle of 27° at one end, enabling easy access to the molars, as depicted in Figure 1. The inner electrode is made of copper and has a diameter of 0.59 mm. On the other hand, the outer electrode is a stainless-steel tube with an internal diameter of 3 mm and an external diameter of 8 mm.

The inner electrode is coated with a dielectric material to create the dielectric barrier discharge. For this purpose, commercial zircon (ZrSiO_4_) powder (zircon potting cement no. 13, Sauereisen, Inc., Pittsburgh, PA, USA) was chosen. This dielectric material exhibits high electrical insulation, thermal conductivity, and anticorrosive properties. When mixed, it forms a chemical setting cement capable of withstanding temperatures above 1400 °C, making it suitable for the plasma conditions and potential temperature gradients and thermal shocks within the reactor.

**Figure 2:**
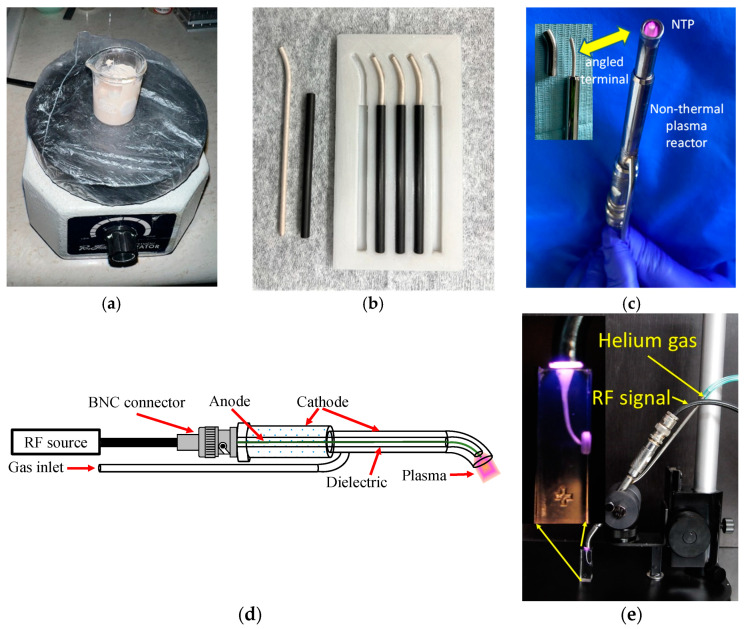
(**a**) Preparation of the mixture for the coating of the central electrode. (**b**) The mixture and corresponding angle cover the central electrode. (**c**) Non-thermal plasma reactor. (**d**) Schematic diagram. (**e**) The NTP reactor test device was applied to a root canal model.

As mentioned earlier, the central copper electrode is coated by mixing 10 g of zircon powder with 1 mL of distilled water. The mixture is degassed using an electric vibrator (Ray Foster Dental Equipment, Huntington Beach, CA, USA) for 2 min, as shown in Figure 2a. To improve adhesion, any linear irregularities on the copper electrode are rectified by rolling it on sandpaper between two flat crystals, thus providing surface roughness.

After injecting the mixture to cover the copper electrode, it is inserted into a silicone hose with an internal diameter of 2.1 mm and an external diameter of 3 mm. The mixture is carefully injected inside the hose until the entire electrode volume is covered. Before the mixture sets, the silicone hose with the covered electrode is introduced into an 80 mm long carbon fibre tube with an internal diameter of 3.1 mm and an external diameter of 5 mm. The silicone hose and carbon fibre tube are placed in a mould to create the required 27° curvature for the reactor design (Figure 2b).

After the zircon mixture has been injected and set around the copper electrode inside the silicone hose and carbon fibre tube, the central electrode, now coated with the zircon material, is carefully retrieved from the hose by making a transverse cut using a scalpel blade. It is then adjusted to the required length to fit inside the reactor (Figure 2c).

The schematic diagram of the entire process is depicted in Figure 2d. Finally, Figure 2e shows the NTP reactor applied to a root canal model, and in the enlarged area of the model, plasma penetration is observed throughout the canal. This demonstrates the successful application of the NTP reactor for dental purposes.

### 2.2. Materials for the Experimentation In Vitro

Several publications have discussed using NTP reactors for treating biofilms [12,31]. Various in vitro tests were conducted to verify the efficiency of the designed device for applying it to patients with root canal problems infected with biofilms. As documented in previous studies, different sampling techniques and identification methods have been employed to examine microbial populations within root canals [32,33]. These studies have revealed the presence of a polymicrobial community within endodontic infections.

The literature reports the presence of various bacteria and microorganisms in root canals [32,34], including Gram-positive bacteria such as *Peptostreptococcus* spp., *Streptococcus* spp., *Enterococcus faecalis*, *Staphylococcus salivarius*, *Lactobacillus* spp., *Actinomyces* spp., *Candida albicans*, *Eubacterium* spp., *Bacillus* spp., and Gram-negative bacteria such as *Porphyromonas* spp., *Prevotella* spp., *Veillonella* spp., and *Escherichia coli* [35], among others. Some species are less prevalent in root canals.

The enduring presence of microorganisms in the apical region of the root canal can potentially result in endodontic diseases, even following the completion of root canal treatment. *Enterococcus faecalis* holds significant relevance in the field, given its widespread presence in cases of endodontic failure. It has been detected in necrotic pulps before and after unsuccessful treatments [34]. This bacterium is inherently highly resistant to antimicrobial drugs compared to other Gram-positive bacteria, making it clinically significant in dentistry. Therefore, for this in vitro study, *Enterococcus faecalis* ATCC 29212 was selected as the bacterium to be investigated.

### 2.3. Construction of Transparent Root Canal Models with Inserted Dentin

This investigation employed a lower second premolar, which had been extracted for orthodontic purposes. Access to the pulp chamber was established, and a type K file was meticulously inserted until it was visible through the apical foramen. The working length was determined by deducting 0.5 mm, and chemomechanical instrumentation was performed using a solution of 5.25% NaOCl and rotary instruments, ultimately achieving a final diameter of 0.45 mm with a taper of 0.06%.

**Figure 3:**
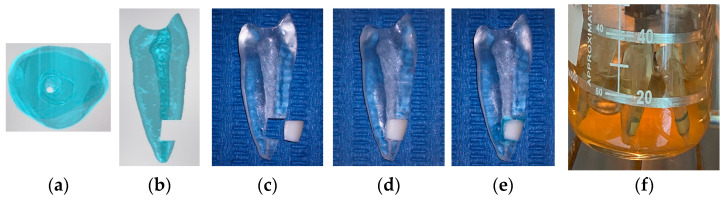
A 3D printed transparent tooth model with the following images: (**a**) top view showing the simulated root canal, (**b**) lateral view, (**c**) presentation of the cast and dentin, (**d**) dentin mounted on the model, (**e**) dentin and cast showing the stamp, and (**f**) biofilm growth.

To obtain 3D images of the premolar, high-definition Planmeca OP300 5 × 5 FOV cone beam, computed tomography (Planmeca, Helsinki, Finland) produced DICOM format images. Using the inVivo5 3D design program (Anatomage, San Jose, CA, USA), a three-dimensional recreation of the premolar was generated in STL format. The design included a 3 mm slot above the apex in its vestibular portion, with dimensions of 3 mm in depth and 3.8 mm in height, ensuring it occupied the entire root canal space. Once the final design was completed, the model was 3D printed using standard transparent biocompatible resin, specifically the 3D AnyCubic 8k with LCD technology (AnyCubic, Shenzhen, China) (see Figure 3a,b).

A 0.45/0.06 rotary file was introduced into the 3D-printed models along their final length to prepare the samples. Additionally, a size 15 K file was passed 0.5 mm through the foramen to ensure the patency of the apical foramen and to check for any resin obstructions in the duct. The samples underwent a 10 min wash with isopropyl alcohol in an ultrasonic bath, and an additional 10 min rinse with distilled water in the same ultrasonic bath.

Human mandibular premolars were carefully selected for this study based on specific criteria, including having a fully formed apex, no root curvature, no fractures, and no signs of resorption. Each tooth had a single root canal. Dentin segments were obtained from these premolars using a diamond disc, and their measurements were precisely matched to the groove in the 3D-printed models. To simulate the root canal space, a standardized round groove with dimensions of 1.0 mm in depth and 1.3 mm in width (Figure 3c) was created at the centre of each dentin specimen using a number 1157 tungsten carbide high-speed bur (SS White, Lakewood, NJ, USA.

To verify the structural integrity of the samples, they were scrutinized under an operating microscope at a 20× magnification level (Opmi Pico, Carl Zeiss, Oberkochen, Germany). Following this preparation, the dentin samples were treated with 17% ethylenediaminetetraacetic acid for 1 min to remove the smear layer, ensuring a clean and consistent surface. Subsequently, the prepared dentin segments were inserted into the designated spaces in the 3D-printed models (Figure 3d). Gingival locking resin (Ultradent Products Inc., South Jordan, UT, USA), was then used to seal the dentin fragments in place securely without causing any damage to the samples (Figure 3e).

Before inoculating and preparing bacterial biofilms, the models shown in Figure 3f underwent gamma irradiation to create a sterile environment. The sterilization procedure involved exposing the model to a dose of gamma rays of 15 kGy, which was emitted from a cobalt-60 radioactive source. This ensured the creation of a sterile environment while preserving the physical integrity of the conduits in the model.

### 2.4. Bacterium Selection and Preparation

The *Enterococcus faecalis* bacterium was cultured in 5 mL of Luria–Bertani (LB) nutrient medium for 20 h at 37 °C. After incubation, the nutrient medium was removed by centrifugation using a Dynamica-brand centrifuge (Dynamica, Livingstone, UK) operating at 5000 rpm for 10 min. This centrifugation process was repeated twice, and, each time, sterile phosphate-buffered saline (PBS) with a pH of 7.2 was used. The supernatants obtained after centrifugation were discarded, and the residues were washed three times with distilled water to eliminate any potential interference from the culture media. Subsequently, the washed residues were resuspended in a PBS solution.

Bacterial counting was conducted using a Carl Zeiss phase-contrast microscope (Carl Zeiss, Oberkochen, Germany) and a Neubauer chamber (Marienfeld GmbH & Co. KG, Lauda-Königshofen, Germany). To guarantee the precision of the bacterial count, serial dilutions at a 1:10 ratio were meticulously prepared and subsequently incubated for 24 h at 37 °C under aerobic conditions. The bacterial cell count in the suspension was determined through the conventional plate count method, and the bacterial cell density was adjusted to 1 × 10^6^ colony-forming units per mL (CFU/mL) and 1 × 10^5^ CFU/mL for subsequent experiments.

### 2.5. Intracanal Biofilm Formation

A single representative colony was chosen to assess the susceptibility of *Enterococcus faecalis* biofilms to NTP, and it was then diluted in 1 mL of sterile PBS. A total of 20 µL of *Enterococcus faecalis* culture was injected into each root canal model containing inserted dentin. Then, 15 mL of Luria–Bertani agar was placed into an 80 mL beaker to introduce the root canal models with inserted dentin until the complete coverage of the specimens with the bacterial suspension up to the dentin of the model was achieved. Each group of specimens consisted of 10 individual models. These groups of specimens were carefully placed in a circular plate made of stainless steel schedule 316L. The circular plate had perforations with a diameter of 0.296 mm, which allowed each model to be securely positioned (Figure 3f).

After the specimens were positioned on the circular plate, they were incubated at 37 °C for three weeks, and this incubation period facilitated the formation and maturation of the biofilm on the dentin surfaces.

The bacterial culture medium was regularly replaced throughout the three-week incubation period. This regular replacement ensured the biofilm had access to a fresh nutritional environment, promoting its continuous growth and development during the study.

### 2.6. Application of Non-Thermal Plasma in the Root Canal

NTP was generated in the plasma reactor, as described in Section 2.1, by utilizing an RF source that operated at a frequency of 13.56 MHz and a power level of 20 W. Helium was employed as the working gas, and it was introduced into the reactor at a flow rate of 0.5 LPM. These parameters resulted in an energy density of 0.50 W/cm^2^ at the tip of the reactor, which is an order of magnitude lower than the recommended exposure limit for individuals according to the International Commission on Non-Ionizing Radiation Protection [36], which is 4 W/cm^2^.

The 3D root canal models from the experimental and control groups were randomly divided for microbiological and SEM analysis. Dentin sections were disassembled from the 3D models after the respective treatments for microbial analysis and transferred to 1 mL tubes. They were then sonicated in a water bath to remove biofilms. Subsequently, samples were diluted from 10^7^ to 10^3^, with 100 μL aliquots from each dilution step of every sample being plated in triplicate on LB agar. Afterward, the plates were left to incubate at 37 °C for 48 h, during which time the resulting colonies were enumerated as CFU/mL.

### 2.7. Generation of Plasma-Treated Water (PTW)

Using the curved-tip reactor, we generated PTW on the surface of sterile distilled water (SDW). The SDW container was positioned on a shaker device to ensure a consistent dispersion of the reactive components within the water. The operational parameters of the NTP reactor remained consistent with those detailed in Section 2.6. The reactor’s tip was positioned at a 3 mm distance from the surface of the SDW within a 5 mL volume, which underwent plasma activation for 10 min. The primary objective of this process was to produce the PTW utilized in the current study.

### 2.8. Experimental Description

**Figure 4:**
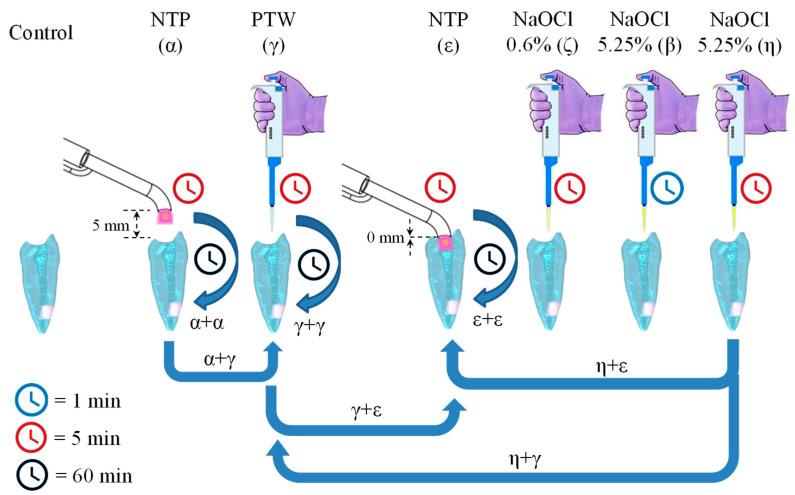
Experimental conditions applied to the 3D models of root canals.

Within the scope of this research, we cultivated biofilms on 3D root canal models containing embedded dentin, as depicted in Figure 3. These biofilms were formed over three weeks, creating a conducive environment for developing and establishing biofilms within the dentin root models. This biofilm formation process was deliberately designed to faithfully emulate conditions observed in real-world clinical settings, thus providing an authentic framework for the subsequent investigation and assessment of treatment outcomes. As illustrated in Figure 4, various combinations were conducted to comprehensively explore and evaluate the effects of multiple treatments on biofilm formation and elimination within environments that faithfully replicate actual clinical conditions. In the experimental phase (refer to Figure 4), the concentration of *Enterococcus faecalis* bacteria in the biofilms was 10^7^ CFU/mL, establishing this as our control condition; in this case, the samples did not undergo any treatment after inoculation, and their initial CFU counts were used as a reference for comparison with the experimental group.

The α condition denoted the application of NTP in the root canal for 5 min, with the reactor’s tip positioned at a 5 mm distance. The γ condition entailed the introduction of PTW into the root canal for 5 min. In another condition, the reactor’s tip was placed directly onto the model, in contact with the dentin, at a distance of 0 mm, designated as the ε condition. Furthermore, we employed NaOCl in 0.6% and 5.25% concentrations. The first concentration was defined as the ζ condition and was applied to the root canal for 5 min, while the second concentration of NaOCl was used for 1 min (β condition) and for 5 min (η condition). All these conditions were considered baseline reference conditions.

In the subsequent stage of experimentation, we replicated the baseline procedures, i.e., α + α, γ + γ, and ε + ε. In each instance, the initial condition was administered, the model with the dentin was refrigerated, and the same procedure was repeated one hour later.

Moreover, we explored specific combinations of the baseline conditions. For instance, we applied NTP treatment (α), immediately followed by the application of PTW (γ) for an additional 5 min, which was referred to as α + γ. Similarly, the combinations were carried out as γ + ε, and, finally, η + γ.

Another phase of the experimentation entailed the combination of the NaOCl at 5.25% (η) and NTP at 0 mm (ε). After applying the NaOCl solution for 5 min, the model with the dentin was refrigerated for 60 min, followed by NTP; we referred to this procedure as η + ε. Lastly, the experiment identified as γ + ε + ε was performed, in which PTW was administered to the model with the dentin for 5 min (γ) without removing the PTW residues. Subsequently, the ε condition was applied, followed by 60 min of refrigeration, and then the ε condition was reapplied for 5 min more.

### 2.9. Scanning Electron Microscopy

We utilized scanning electron microscopy (SEM) and energy-dispersive X-ray microanalysis (EDX) to examine zircon powder during this study. Additionally, SEM was used independently to investigate the ultrastructural and physicochemical characteristics of the formed biofilm, and the morphology of both the biofilms and microorganisms within the dentin treatment areas.

Dentin samples were collected from the treated and control root canal models and prepared for SEM analysis using the following described methodology. The samples were harvested, washed, and fixed with glutaraldehyde in solution at 6% in 0.1 M of PBS and placed under refrigeration at 4 °C for 24 h. Samples were then postfixed in osmium tetroxide (solution at 4%) in 0.1 M of PBS and refrigerated at 4 °C for 2 h. Finally, the samples were rinsed with PBS and dehydrated in ascending graded ethanol series (20, 30, 40, 60, 70, 80, 90, and 100%). The samples were subsequently affixed to be mounted on a stub and then subjected to an ion sputter process to apply an ultra-thin layer of gold.

To perform the SEM analysis, we utilized a scanning electron microscope model JEOL JSM 5900 (JEOL Ltd., Tokyo, Japan), with the following radiation parameters: high voltage of 20 kV and a probe current of 25 pA. These settings provided the necessary resolution and sensitivity to examine the surface morphology and composition of the biofilm, enabling us to gain valuable insights into its structure and interactions with the dentin surfaces.

### 2.10. Statistical Analysis

The data analysis was conducted using two software tools: a spreadsheet program (Excel 97, Microsoft Corp., Richmond, VA, USA) and Origin-Pro software ver. 8.0 SR2 (OriginLab Corporation, Northampton, MA, USA). Statistical tests, including the ANOVA with Bonferroni and Tukey’s post hoc tests, were performed at a significance level of 5%. We rigorously analysed and interpreted the experimental results by applying these statistical methods, deriving meaningful conclusions from the data.

## 3. Results

### 3.1. Results of the Non-Thermal Plasma Reactor with a Curved Terminal

**Figure 5:**
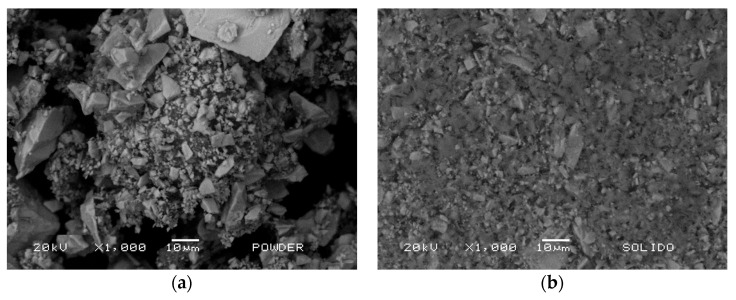
(**a**) SEM micrograph of the raw powder. (**b**) SEM micrograph of the processed powder.

The analysis of the zircon powder was conducted to evaluate the composition of the dielectric using a scanning electron microscope JEOL JSM 5900, operating at an electron energy of 20 kV. Figure 5a displays SEM micrographs of the zircon-based powder, revealing a dispersed crystal form, the characteristics of which are detailed in Table 1. In Figure 5b, the solid structure of the central electrodes of the DBD reactor covered with the zircon coating is presented, and its features are detailed in Table 1. The uniformity of the powder used across the entire surface can be observed after being heated at 1200 °C. This uniform coating is crucial to ensure the desired effect of the DBD in the plasma reactor. 

A Siemens D5000^®^ CuKa diffractometer (Siemens Corp., New York, NY, USA) was utilized to determine the crystalline phases in the zircon powder used to coat the central electrode. The diffractometer was operated with an acceleration set at 35 kV and a current of 25 mA. Diffractograms were obtained in the 15–80° (2θ) range using a step size of 0.02°, and each step took approximately 38.9 s.

**Figure 6:**
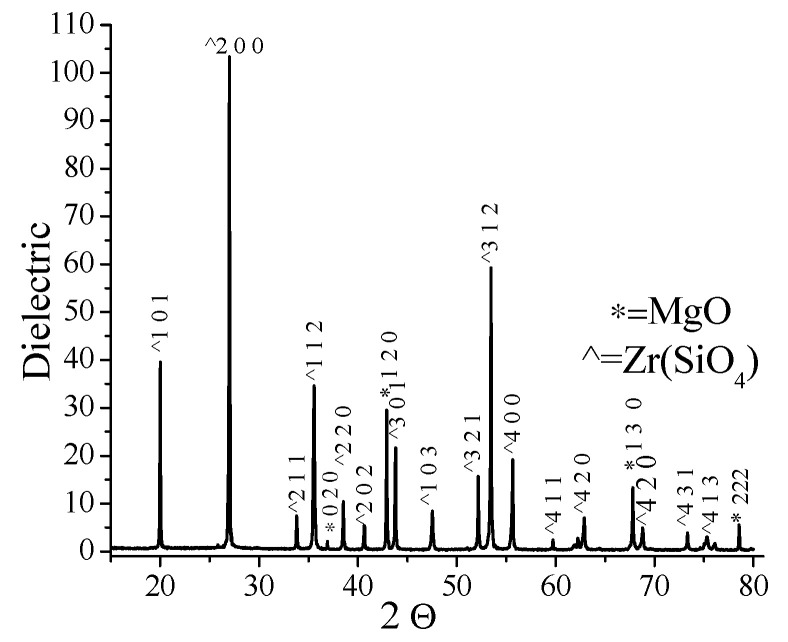
Diffractogram of the powder used for the coating of the central electrode.

The X-ray diffraction (XRD) results were analysed by comparing them with reference standards from the ICDD (International Centre for Diffraction Data) database using DIFFRAC.EVA V4.1.1 software (Bruker, Billerica, MA, USA). Figure 6 displays the corresponding XRD diffractogram, which indicates the presence of zircon and magnesium oxide in the powder. 

The thermal treatment of the zircon powder during the coating of the central electrode did not lead to substantial changes in peak widths, indicating no significant alterations in the crystal lattice structure.

The NTP generated by the curved-tip reactor produced a significant concentration of reactive species. In particular, the generated NTP provides an optical emission spectrum (OES) identical to that observed in previous works [37,38], where primary reactive oxygen species (ROS) were identified, such as the hydroxyl radical (OH), the nitric oxide (NO), and singlet oxygen (^1^O_2_), which give rise to the formation of secondary species, including molecular oxygen (O_2_), hydrogen peroxide (H_2_O_2_), atomic oxygen (O), and ozone (O_3_). Some of these primary species have a half-life of the order of microseconds [39]. Furthermore, various species were identified because NTP is generated with helium gas, such as the molecular nitrogen ion (N_2_^+^) and helium atomic radicals. The presence of the NOγ(A-X) system was also determined in the spectrum, which results from the dissociation of the surrounding air and the subsequent formation of NO molecules from oxygen (O) and nitrogen (N) atoms.

Secondary reactive species can persist from milliseconds to several days [40]. In summary, the generation and evolution of reactive species produced by NTP represent a complex and dynamic process that involves multiple stages, transitioning from gas to liquid phases. This profound understanding of the underlying chemistry carries significant implications for microbial inactivation.

### 3.2. NTP Temperature Measurement

In biomedicine, the temperature generated in the NTP is critical and should not exceed 40 °C to avoid the thermal damage and destruction of living tissue [41]. In this study, the NTP beam was directed onto a glass plate where a type K thermocouple was placed for temperature measurements. The outlet nozzle of the reactor was varied perpendicular to the glass plate between 1 and 10 mm, with the specific power of the RF generator set between 16 and 24 W and a constant helium gas flow rate of 0.5 LPM.

**Figure 7:**
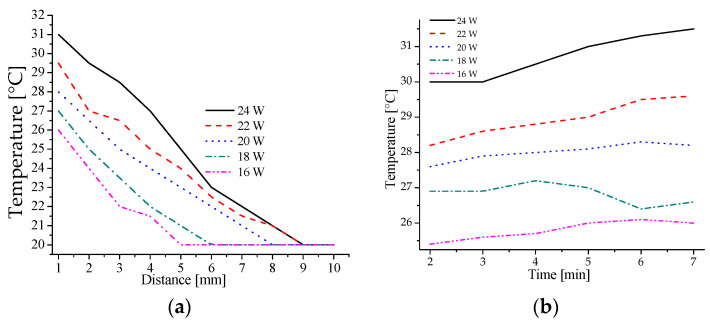
Temperature measurements with two graphs: (**a**) effect of applied power concerning distance; (**b**) effect of applied power concerning time.

Subsequently, at a fixed distance of 5 mm, the plasma application time was varied between 2 and 7 min (see Figure 7). These conditions were tested for their effectiveness in eliminating bacteria, and the results are presented in Figure 6 and Figure 7a. For each data point, five samples were averaged, and the average of these measurements was reported as the result. Figure 7a,b exemplify that with 20 W of power and a five-minute application time, the sample’s temperature is maintained at a reasonable 28 °C, making it suitable for application to patients and ensuring no thermal damage to biological tissue.

### 3.3. Experimentation with the Reactor with Bacteria and Biofilms In Vitro

**Figure 8:**
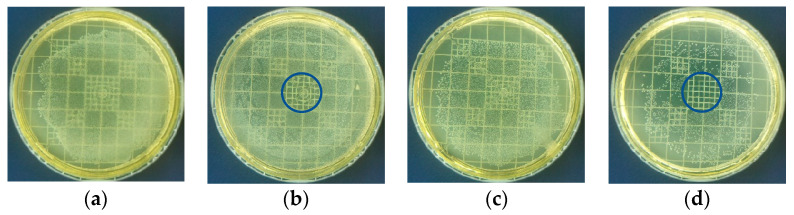
The inactivation of *Enterococcus faecalis* bacteria under various conditions. (**a**) Control at 10^6^ CFU/mL. This image illustrates bacterial growth without any treatment. (**b**) Concentration of 10^6^ CFU/mL treated with NTP. The treated area is highlighted by a circle, demonstrating bacterial inactivation. (**c**) Control at 10^5^ CFU/mL. This image displays bacterial growth without NTP treatment. (**d**) Concentration of 10^5^ CFU/mL treated with NTP. Once again, the treated area is marked with a circle, showcasing bacterial inactivation at this concentration.

Experiments were conducted initially in Petri dishes and subsequently on root canal models with embedded dentin to assess the time required for *Enterococcus faecalis* bacterial inactivation. Figure 8 illustrates the outcomes of in vitro bacterial inactivation at 10^6^ and 10^5^ CFU/mL concentrations, employing a 5 min NTP application while maintaining a 5 mm distance between the reactor tip and the plate. In Figure 8, the region where the plasma was applied is delineated by a circle, covering an approximate area of 1 cm².

We conducted bacterial inactivation using PTW on the same strain in the subsequent phase. Water distilled with a volume of 5 mL was utilized, and the treatment duration was set to be 5 min. Water characterization revealed a pH of 4.3, ozone concentration of 0.8 ppm, and a hydrogen peroxide concentration of 3 ppm. Employing PTW, *Enterococcus faecalis* was exposed to the bacteria at a 10^6^ CFU/mL concentration, leading to complete bacterial inactivation within 5 min.

### 3.4. Experimentation of the Reactor with Bacteria and Biofilm Root Canals

The experimental phase aimed at removing biofilms from root canals followed the protocol outlined in Section 2.8 and depicted in Figure 4. Biofilms were cultivated within root canal models containing embedded dentin, as illustrated in Figure 3. The formation of these biofilms spanned three weeks (see Section 2.5), enabling bacterial proliferation and the establishment of biofilms within the dentin. This biofilm formation process replicates conditions encountered in clinical practice, providing a realistic framework for research and subsequent treatment evaluation.

**Figure 9:**
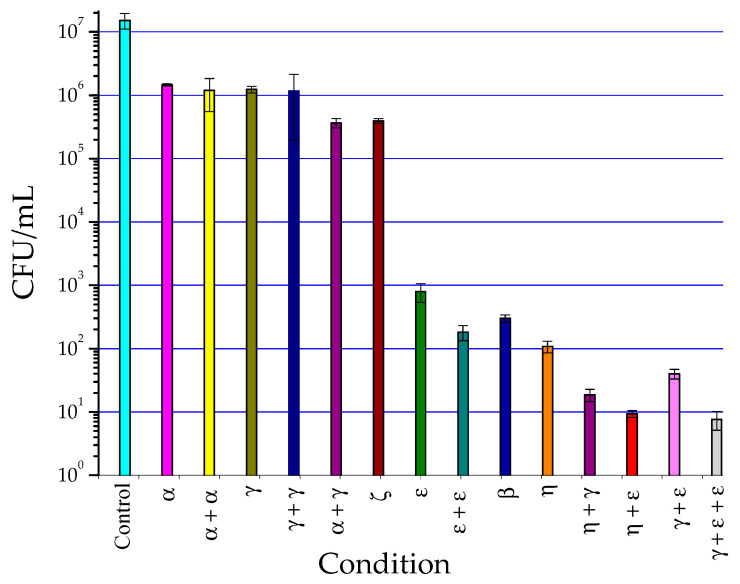
Survival graph of biofilms in the biomodels under different conditions.

The concentration of *Enterococcus faecalis* bacteria in the control model was consistent at approximately 1.52 × 10^7^ CFU/mL, as depicted in Figure 9 and Table 2. A comparative analysis was conducted among the NTP, PTW, and NaOCl at 5.25% procedures, along with the gold standard NaOCl at 0.6%, as detailed in Table 2. This table delineates the conditions under which the processes exhibited statistical significance (value 1, *p* < 0.001) and those where significance was not observed (value 0, *p* = 0.999).

These findings emphasize the importance of treatment combinations and their sequencing to achieve effective inactivation of *Enterococcus faecalis* within root canals. Additionally, they provide valuable insights into the effectiveness of different approaches and potential limitations in this specific application. These results contribute to advancing therapeutic strategies in biofilm removal in endodontic procedures.

**Figure 10:**
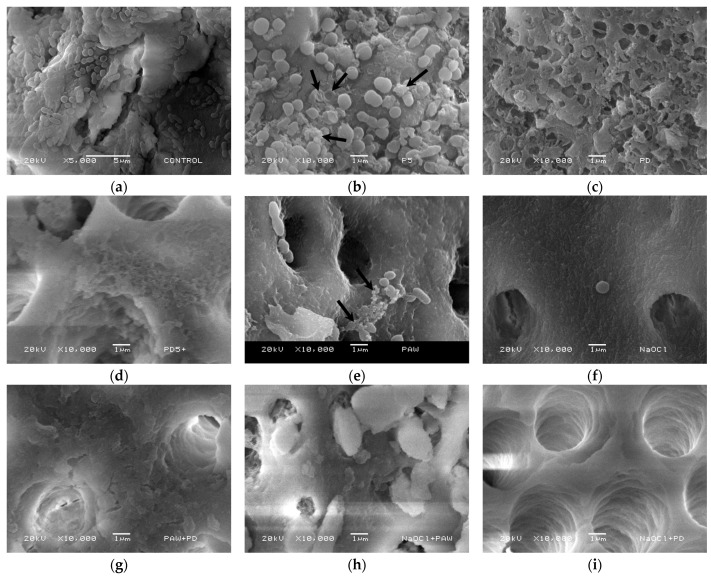
Longitudinal section of root canal analysed by SEM: (**a**) Dentin fully colonized by *Enterococcus faecalis.* Biofilms under various treatment conditions: (**b**) α, (**c**) ε, (**d**) ε + ε, (**e**) γ, (**f**) η, (**g**) γ + ε, (**h**) η + γ, and (**i**) Treatment η + ε. Arrows indicate changes in cellular morphology, alterations, and debris.

We utilized SEM to investigate how different treatments affected the morphology of bacteria attached to the dentin surface (Figure 10). The *Enterococcus faecalis* biofilm in Figure 10a appears to adhere to and colonize the sample’s surface, with generally intact cell membranes. NTP treatment led to some *Enterococcus faecalis* cells displaying a deflated and ruptured morphology, accompanied by debris, as indicated by arrows in Figure 10b. The cell membranes became considerably rough and unstructured in samples where the NTP was applied at a 0 mm distance from the nozzle (Figure 10c). Similar results were observed when the NTP was used for 5 min and then again after a 60 min interval (Figure 10d). The treatment with PTW (Figure 10e) reduced the presence of these microbes in the sample; however, some cells were still visible, and, among them, specific cells exhibited ruptured or damaged cell walls, as indicated by the arrows. In the case of NaOCl treatment (Figure 10f), the canal walls appeared clean, and the presence of cells was minimal. When combined treatments were applied, such as PTW and NTP at a 0 mm distance from the nozzle (Figure 10g) and NaOCl and PTW (Figure 10h), the number of attached cells on the surface rapidly decreased, and ruptured cell membranes were observed.

## 4. Discussion

Emerging microbial control technologies offer numerous advantages in the scientific field, including the preservation of oral health. Microbial load and the virulence of various oral microorganisms significantly impact microbial contamination assessment, with specific pathogens posing risks in root canals. Over the decades, innovative techniques have been developed to address oral health challenges. However, it is essential to note that some decontamination methods may adversely affect oral properties.

Combining multiple treatment methods, especially for root canals, has shown promise by synergistically combating oral health-threatening microorganisms, providing effective alternatives for achieving desired outcomes. Although this multidisciplinary approach creates an inhospitable environment for microorganisms, a definitive solution still needs to be discovered. These approaches aim to optimize therapeutic interventions and enhance patients’ quality of life, contributing to ongoing dental advancements and promoting optimal oral health.

This study aimed to develop a non-thermal plasma generator reactor with a curved terminal and assess its performance in the inactivation of biofilms in Petri dishes and root canals. Furthermore, the coating exhibited no detectable porosities, ensuring electrical insulation and the effective operation of the plasma reactor. The absence of porosities is critical to maintaining electrical integrity and preventing any potential plasma leakage, thereby contributing to the overall success of the plasma reactor in its intended applications. Traditional plasma generators typically possess rigid and straight macroscopic characteristics. To initiate the discharge, the polarization of the two electrodes is achieved using a high-voltage source in the order of kilovolts, employing alternating or pulsed current with an operating frequency in the tens of kilohertz range. The gas flow rate during the experimentation was consistently maintained at approximately 10 LPM [42,43,44,45,46,47,48]. The electrical properties resemble those of curved-type reactors, such as plasma jets [28,29], or the Thorns plasma reactor [30]. In our previous work, we designed and constructed a reactor with a straight output, which has found applications in clinical settings for treating patients with burns [38], addressing oral health issues [49,50,51,52], managing diabetic foot conditions [53,54], and performing neck surgeries [55].

The power supply characteristics used in these applications included a voltage range of ±200 V, a frequency of 13.56 MHz, and a gas flow rate of 0.5 LPM [56], significantly reducing the electrical characteristics at which the reactor operates. Bacterial inactivation studies conducted with the curved-output NTP reactor demonstrated excellent bacterial inactivation. These disinfection studies highlight the reactor’s potential for root canal treatment. Furthermore, during its use, the proposed curved-output NTP reactor does not significantly increase the heat on the surface where the plasma is applied, making it very useful in preventing heat damage to dental pulp [57].

Many research endeavours have been dedicated to unveiling the bacterial species inhabiting root canals [58,59,60,61]. A substantial body of literature has documented the effective inactivation of bacteria through NTP treatment, underscoring its potent antibacterial efficacy [44,60,62]. The results consistently demonstrate complete bacterial inactivation within the treated area, primarily attributed to the influence of reactive oxygen and nitrogen species, a phenomenon that has been observed by other researchers as well [41]. These seminal findings provided a solid foundation for the subsequent application of NTP in root canals. Furthermore, encouraging outcomes have been reported regarding the in vitro eradication of the Gram-positive Enterococcus faecalis using reactors featuring curved outlets [28,29]. These reactors typically operate under high voltages and frequencies in the tenths of kilohertz range. In contrast, our proposed reactor sets itself apart by employing lower voltages and frequencies in the megahertz range, representing a significant departure from established methodologies in the field.

Chronic periradicular lesions, both before and after root canal therapy, have garnered significant attention in the literature [12,35]. Biofilms have proven to be more resistant to antimicrobial agents than planktonic bacteria. Given this challenge, *Enterococcus faecalis* was chosen as the model organism for our in vitro studies, representing endodontic disinfection investigations. Preclinical and clinical studies have reported that the separate application of NTP and PTW promotes anti-inflammatory and microbicidal functions, facilitating tissue repair and contributing to the restoration of periodontal tissue. The extent of this recovery is contingent on the number of applications, as supported by research such as [49,50,51,52,63,64].

The outcomes of our experiments involving *Enterococcus faecalis* and its treatment with NTP and PTW using an innovative reactor with a curved terminal underscore a remarkable level of success. This achievement can be attributed to our meticulous examination of the distinct phases involved in the inactivation process of *Enterococcus faecalis*. These findings emphasize the importance of treatment combinations and their sequencing in achieving *Enterococcus faecalis* inactivation within root canals, offering valuable insights into the effectiveness of different approaches for this specific application.

*Enterococcus faecalis*, a bacterium known for its resistance to conventional sterilization methods, including heat, acid, calcium hydroxide, and UV irradiation [65], has posed a significant challenge in root canal treatments. The combined experiments conducted in this study unveil several effective strategies for enhancing the inactivation of *Enterococcus faecalis* within root canals. These experiments clearly illustrate the efficacy of treatment combinations involving NTP, PTW, and NaOCl applied under various sequences and conditions. In our study, the microbiological results strongly indicate the susceptibility of *Enterococcus faecalis* to the NTP and PTW generated by our proposed reactor, with noticeable effects on *Enterococcus faecalis* biofilms becoming apparent after just 5 min of treatment. Plasma exposure rapidly disrupts the cell wall, as visually depicted in Figure 10, leading us to infer that the cell membrane incurred damage from the NTP [66]. This compromised membrane renders microorganisms highly vulnerable to the reactive environment, facilitating the penetration of reactive oxygen and nitrogen species, photons, and charged particles, ultimately reaching the DNA material [67]. It is this mechanism that underscores the effectiveness of our NTP-based approach in inactivating Enterococcus faecalis, providing a promising avenue for improved root canal sterilization.

The colonization of *Enterococcus faecalis* bacteria was also studied using SEM. These results revealed distinct differences in root canals, with significant effects observed when using NTP and PTW treatments, especially when combined, compared to the individual use of sodium hypochlorite. Notably, the combined treatment of NaOCl and NTP at a 0 mm distance from the nozzle (Figure 10i) demonstrated more effective microbial detachment. These findings and microbiological analysis demonstrate the promising potential of these mixed methods in oral treatments, particularly for root canals. These approaches synergistically combat oral health-threatening microorganisms, providing adequate alternatives. While a definitive solution remains elusive, these multidisciplinary approaches aim to enhance therapeutic interventions and improve patients’ quality of life, contributing to dental advancements and optimal oral health promotion. In the studies reported by Kerlikowski and collaborators [68], they suggested the design and construction of a reactor that would facilitate the application of the NTP, and we consider that compliance is achieved with the proposed reactor. It is worth noting that treatments with our innovative curved-tip reactor involve perpendicular application to the 3D model, achieving a similar effect to that offered by the researchers mentioned above.

## 5. Conclusions

In this study, we introduce an innovative reactor design with a curved termination for generating non-thermal plasma (NTP) and plasma-treated water (PTW) to inactivate biofilms in root canals. The reactor utilizes zircon (ZrSiO_4_) powder coating on the central electrode, ensuring stable NTP production suitable for dental applications, particularly in root canal treatments. The curved-terminal reactor offers an ergonomic and efficient solution for accessing root canals, applying NTP, and inactivating biofilms of *Enterococcus faecalis*. The combined treatments involving NaOCl, NTP, and PTW exposure demonstrated significantly heightened antimicrobial efficacy compared to the control group. The unique antimicrobial efficacy of NTP and PTW is attributed to their ability to disrupt microbial structures, including those within biofilms. This breakthrough can revolutionize root canal disinfection, providing a superior and optimized approach. In summary, our study represents a substantial advancement in dentistry, presenting an innovative method for biofilm inactivation in root canals using NTP generated by a curved-terminal reactor. Implementing this technology can improve the prevention and management of endodontic infections. Further research with this reactor design is essential for complete bacterial inactivation in root canals.

## Figures and Tables

**Table 1:** Composition characteristics of zircon before and after processes.

Element	Raw Powder [at.%]	Processed Powder [at.%]
C	63.70	40.71
O	25.10	39.31
Mg	0.39	6.22
Si	5.16	3.82
Zr	5.25	3.28
Al	0.31	-
P	-	6.66

**Table 2:** Comparison with NaOCl “the gold standard”.

Treatment	log_10_ CFU/mL	*p* Value versus ζ	Significance *
ζ	5.59 ± 0.03	Without value	Without value
Control	7.17 ± 0.11	0.999	0
α	6.16 ± 0.01	0.999	0
α + α	6.02 ± 0.25	0.999	0
γ	6.09 ± 0.05	0.999	0
γ + γ	5.87 ± 0.48	0.999	0
α + γ	5.56 ± 0.07	0.999	0
ε	2.88 ± 0.16	<0.001	1
ε + ε	2.15 ± 0.21	<0.001	1
β	2.47 ± 0.06	<0.001	1
η	2.03 ± 1.36	<0.001	1
η + γ	1.26 ± 0.10	<0.001	1
η + ε	0.97 ± 0.06	<0.001	1
γ + ε	1.60 ± 0.08	<0.001	1
γ + ε + ε	0.87 ± 0.15	<0.001	1

* The significance level is 1 if the difference to NaOCl 0.6% is significant at 0.05 and 0 if it is not significant at the same level.

## Data Availability

Data are contained within the article.
